# Efficacy and safety of the third-generation tyrosine kinase inhibitor Olverembatinib in relapsed and persistent minimal residual disease positive Philadelphia chromosome-positive acute lymphoblastic leukemia patients

**DOI:** 10.3389/fmed.2025.1662512

**Published:** 2025-10-03

**Authors:** Xinwen Jiang, Minran Zhou, Junjie Ma, Qingli Ji, Xiaoqing Li, Sai Ma, Chunyan Chen

**Affiliations:** ^1^Department of Hematology, Qilu Hospital of Shandong University, Jinan, Shandong, China; ^2^Department of Hematology, Yantai Yuhuangding Hospital, Yantai, Shandong, China

**Keywords:** acute lymphoblastic leukemia, Philadelphia chromosome-positive, relapsed, MRD-positive, tyrosine kinase inhibitor

## Abstract

**Objective:**

This study aimed to evaluate the efficacy of Olverembatinib in patients with relapsed and persistent minimal residual disease-positive Philadelphia chromosome-positive acute lymphoblastic leukemia.

**Methods:**

We conducted a retrospective analysis of clinical characteristics in 22 patients diagnosed with Ph + ALL at Qilu Hospital of Shandong University and Yantai Yuhuangding Hospital between December 2018 and December 2024.

**Results:**

A cohort of 22 patients with Ph + ALL was enrolled in this study. Among them, 12 (54.5%) patients had relapsed Ph + ALL, while the remaining 10 patients exhibited persistent MRD positivity. In the relapsed subgroup, the CR rate following one treatment cycle reached 75.0% (9/12), with MRD-negative and MMR rates of 75.0% (9/12) and 50.0% (6/12), respectively. Upon completion of two treatment cycles in evaluable patients (*n* = 8), the CR, MRD-negative, and MMR rates all rose to 87.5, 87.5 and 87.5%, respectively. The patients with forfeited MMR on first or second-generation TKIs, of which the MMR rate was restored to 60.0% (6/10) after one cycle of Olverembatinib treatment, and a pleasant surprise was that their MMR rate soared to 90% (9/10) after two cycles of Olverembatinib treatment. 70% of them subsequently underwent successful hematopoietic stem cell transplantation.

**Conclusion:**

The efficacy and tolerability of Olverembatinib were confirmed in patients with relapsed, MRD-positive, Ph + ALL, offering a novel therapeutic approach for these patients and making prolonged survival possible.

## 1 Introduction

The formation of the Philadelphia chromosome is a significant molecular hallmark of Ph + ALL patients (1). In the pre-TKI era, literature reported that Ph + ALL patients achieved a 5-year overall survival rate of no more than 30% following chemotherapy (2). With the introduction of first-generation TKI (imatinib) combined with chemotherapy, studies documented an improved 5-year overall survival rate of 40–45% in Ph + ALL patients (3, 4). As drug research advanced, third-generation TKI (ponatinib) combined with hyper-CVAD therapy achieved a remarkable 5-year overall survival rate of 75%, significantly enhancing the outcome of Ph + ALL patients (5). In a phase III clinical trial, Jabbour et al. used ponatinib or imatinib in combination with reduced-intensity chemotherapy to treat newly diagnosed Ph + ALL patients (6). After 20 cycles of treatment, the MRD-negative complete remission rate (primary end point) was significantly higher with ponatinib (34.4% [53/154]) vs. imatinib (16.7% [13/78]) (risk difference, 0.18 [95% CI, 0.06–0.29]; *p* = 0.002). Ponatinib demonstrated a superior rate of MRD-negative complete remission at the end of induction vs. imatinib. However, ponatinib was associated with severe cardiovascular adverse events (7), leading to its inclusion in the U.S. FDA’s warning.

Olverembatinib, China’s original third-generation TKI, is indicated for CML patients in chronic or accelerated phases who develop resistance to any TKI or harbor the T315I mutation. Jiang Q et al. (8) reported that CML-CP patients achieved an 85.6% 5-year progression-free survival rate after Olverembatinib treatment. Zhu, Y et al. (9) enrolled 20 newly diagnosed Ph + ALL patients who were administered an Olverembatinib-based regimen as first-line therapy (for the induction treatment, 14 patients received Olverembatinib combined with chemotherapy of VP, 4 with blinatumomab, and 2 with prednisone alone). CR was achieved in all patients, and 85% of the cohort attained CMR with the incorporation of blinatumomab. In a single-center observational study, 14 Chinese patients with relapsed/refractory (R/R) Ph + ALL were enrolled (10). 71.4% achieved an overall response, and the median event-free survival and overall survival were 3.9 and 8.3 months, respectively. Nevertheless, limited data exist regarding Olverembatinib’s efficacy in Ph + ALL patients. This study presents the multi-center clinical data of Olverembatinib-treated Ph + ALL cases.

## 2 Materials and methods

### 2.1 Patient cohorts

This was a retrospective, multi-center study for relapsed and persistent MRD-positive Ph + ALL patients who received Olverembatinib treatment in Shandong province from December 2018 to December 2024. For relapsed and persistent MRD-positive patients, they had previously achieved complete remission (CR) after receiving first-generation or second-generation tyrosine kinase inhibitor (TKI) drugs in combination with chemotherapy. During the maintenance phase, they only took oral first-generation or second-generation TKI medications. However, in the maintenance phase, some patients had more than 5% bone marrow blasts at the time of re-examination, and the other patients had continuous positive MRD at the time of re-examination.

### 2.2 Methods

SPSS software (version 25.0) was used for the statistical analysis. Count data were expressed as cases or rates. For measurement data that conformed to the normal distribution, these were expressed as mean ± SD, and for measurement data that did not conform to the normal distribution, the median (interquartile range) was used. The median Overall Survival (OS) was estimated using the Kaplan–Meier method.

### 2.3 Definition

Complete Response (CR): No blasts in peripheral blood, absence of extramedullary leukemia; restoration of trilineage hematopoiesis in bone marrow with blasts <5%; absolute neutrophil count ≥1.0 × 10^9^/L; platelet count ≥100 × 10^9^/*L. major* Molecular Response (MMR): BCR-ABL1 transcript level measured by qPCR ≤0.1%. Complete Molecular Response (CMR): BCR-ABL1 transcript level measured by qPCR ≤0.01%. Overall Survival (OS): Time from diagnosis to death or last follow-up. All cases were followed up until October 31, 2024. Treatment-emergent adverse events (TEAEs) were graded according to the National Cancer Institute’s Common Terminology Criteria for Adverse Events (CTCAE) v5.0.

## 3 Results

### 3.1 Patient characteristics

A total of 22 patients were included in this study (Table 1): 12 patients with relapsed Ph + ALL and 10 patients with persistent MRD positivity and loss of MMR. Relapsed group: Among the 12 patients (4 males/8 females), the median age was 53.0 years (42.0–57.0). Before Olverembatinib treatment, median values were: White blood cell count: 9.5 × 10^9^/L (range: 5.2–25.0 × 10^9^/L); Hemoglobin: 111.0 g/L (range: 93.0–118.0 g/L); Platelet count: 150.0 × 10^9^/L (range: 120.5–316.0 × 10^9^/L); 75.0% (9/12) of patients exhibited the BCR-ABL1 P190 transcript; Genetic mutations: T315I mutation in 5 patients, IKZF1 mutation in 1 patient. MRD positive group: Among the 10 patients (4 males/6 females), the median age was 48 years (37.0–52.0). Median values were: White blood cell count: 4.3 × 10^9^/L (range: 2.7–5.9 × 10^9^/L); Hemoglobin: 103.5 g/L (range: 87.0–126.0 g/L); Platelet count: 225.0 × 10^9^/L (range: 157.0–305.0 × 10^9^/L); 70.0% (7/10) of patients exhibited the BCR: ABL1 P190 transcript; Genetic mutations: T315I mutation in 1 patient, IKZF1 mutation in 1 patient, and E255K mutation in 1 patient.

**Table 1:** Baseline characteristics of 22 Ph + ALL patients.

Patient	Gender	Age (years)	BM (%)	MFC (%)	WBC 10^9^/L	HGB g/L	PLT 10^9^/L	BCR-ABL1 transcript	ABL1 mutation	BCR-ABL/ABL (IS%)
1	Female	47	61	43.44	4.54	110	150	P190	T315I	163.7
2	Female	57	80	52.71	62.42	66	765	P190	T315I	556.9
3	Female	52	81	84.96	9.43	118	122	P190	N	49.74
4	Male	53	89	74.58	38.72	97	43	P190	T315I	258.9
5	Female	57	19	23.50	5.91	118	320	P190	N	42.43
6	Male	54	73	80.80	23.35	128	90	P210	T315I IKZF1	111.3
7	Female	37	44	25.70	9.52	110	220	P210	T315I	2.28
8	Male	35	7	11.37	4.97	135	143	P190	N	42.2
9	Female	40	84	67.35	6.78	112	317	P190	N	109.4
10	Female	58	6	2.65	2.64	91	120	P210	N	64.0
11	Female	66	12	12.62	11.84	118	150	P190	N	14.0
12	Male	49	26	15.5	11.96	69	314	P190	N	125.59
13	Female	53	0	0.1	1.77	96	200	P190	N	0.44
14	Male	30	0	<0.01	2.85	133	158	P210	N	3.31
15	Female	50	0	<0.01	5.38	132	299	P190	IKZF1	3.74
16	Female	19	0	<0.01	7.28	119	416	P190	T315I	0.21
17	Female	39	0	<0.01	4.35	85	67	P210	N	2.65
18	Female	72	0	<0.01	4.77	108	225	P190	E255K	6.29
19	Male	40	0	<0.01	3.12	124	153	P190	N	5.7
20	Male	52	0	<0.01	4.19	83	225	P190	N	0.21
21	Male	49	0	<0.01	9.04	99	248	P210	N	0.14
22	Female	47	0	<0.01	2.11	88	323	P190	N	2.1

BM, blast cells in bone marrow; MFC, proportion of blast cells by flow cytometry; ABL1 mutation, take the patient’s bone marrow sample for next-generation gene sequencing; N, no ABL1 mutation.

### 3.2 Efficacy

**Figure 1:**
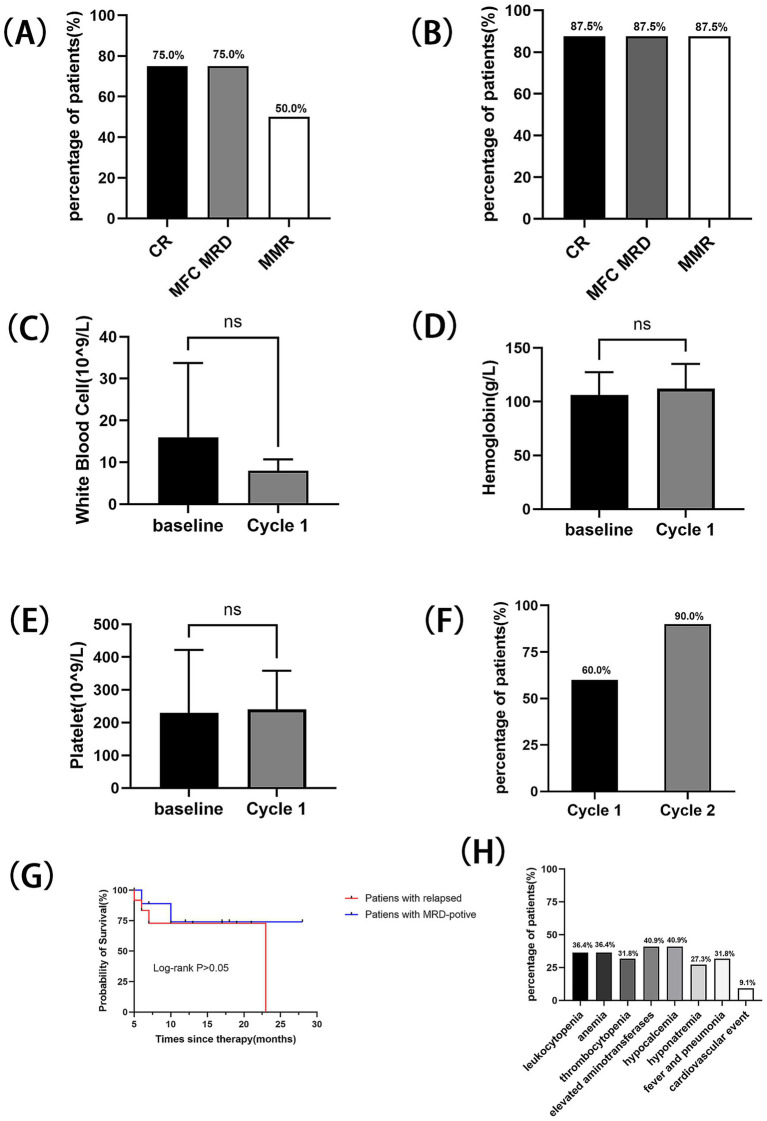
Outcome with the treatment of Olverembatinib in two subgroups: **(A)** CR, MRD-negative, and MMR rates after one treatment cycle for relapsed patients. **(B)** CR, MRD-negative, and MMR rates after two treatment cycles for relapsed patients. **(C)** Changes in white blood cell count following one cycle of Olverembatinib treatment in the relapsed treatment group. **(D)** Changes in hemoglobin level following one cycle of Olverembatinib treatment in the relapsed treatment group. **(E)** Changes in platelet count following one cycle of Olverembatinib treatment in the relapsed treatment group. **(F)** MMR rates after the first and second treatment cycles in the MRD-positive subgroup. **(G)** OS for two subgroups. **(H)** Adverse effects for all patients.

A cohort of 22 patients with Ph + ALL was enrolled in this study. Among them, 12 (54.5%) patients had relapsed Ph + ALL, while the remaining 10 patients exhibited persistent MRD positivity. The treatment plans for these patients and the therapeutic effects after each treatment cycle were recorded (Table 2). In the relapsed subgroup, the CR rate following one treatment cycle reached 75.0% (9/12), with MRD-negative and MMR rates of 75.0% (9/12) and 50.0% (6/12), respectively (Figure 1A). Upon completion of two treatment cycles in evaluable patients (*n* = 8), the CR, MRD-negative, and MMR rates all rose to 87.5, 87.5 and 87.5%, respectively (Figure 1B). There was no significant difference in blood routine examinations compared to baseline levels (Figures 1C–E), and the median OS was 23 months (Figure 1G). The patients with forfeited MMR on first or second-generation TKIs, of which the MMR rate was restored to 60.0% (6/10) after one cycle of Olverembatinib treatment, and a pleasant surprise was that their MMR rate soared to 90% (9/10) after two cycles of Olverembatinib treatment (Figure 1F). 70% of them subsequently underwent successful hematopoietic stem cell transplantation, with the median OS not yet reached (Figure 1G).

**Table 2:** The outcome of treatment with Olverembatinib.

Patient	Cycle 1	BM (%)	MFC (%)	ABL (%)	Cycle 2	BM (%)	MFC (%)	ABL (%)
1	Olv + VIP	0	<0.01	0	Olv + VCP	0	<0.01	2.9*10^−7^
2	Olv + DCME	9	72.71	73.51	Olv + VCP	72	4.86	56.30
3	Olv + CP	0	<0.01	0	Olv + Bli	0	<0.01	0
4	Olv + Bli	0	<0.01	0	Olv + Bli	0	<0.01	0
5	Olv + VP	2	<0.01	2.45	Olv + VP	0	<0.01	0
6	Olv + VP	50	38.97	41.04	N	N	N	N
7	Olv + CVAD	0	<0.01	1.44	Olv + MA	0	<0.01	0.04
8	Olv + VD	0	<0.01	0.003	N	N	N	N
9	Olv + VP + Bli	0	<0.01	0.13	N	N	N	N
10	Olv	4	1.31	26.5	N	N	N	N
11	Olv + VCP	0	<0.01	0.05	Olv	0	<0.01	0.02
12	Olv + VP	0	<0.01	0.08	Olv + Bli	0	<0.01	0.003
13	Olv + IO	0	<0.01	0.037	Olv	0	<0.01	<0.01
14	Olv + Bli	0	<0.01	0	Olv	0	<0.01	0
15	Olv	2	<0.01	0.34	Olv	0	<0.01	0.011
16	Olv + Bli	0	<0.01	0.1	Olv	0	<0.01	0
17	Olv + MTX	0	<0.01	0.52	Olv	0	<0.01	0
18	Olv + VP	0	<0.01	1.2	Olv + VP	0	<0.01	0
19	Olv + Bli	0	<0.01	0	Olv + Bli	0	<0.01	0
20	Olv + Bli	0	<0.01	0.01	Olv	0	<0.01	<0.01
21	Olv	0	<0.01	0.32	Olv + Bli	0	<0.01	0.12
22	Olv	0	<0.01	<0.01	Olv	0	<0.01	<0.01

Olv, Olverembatinib; Bli, blinatumomab; MA, Methotrexate + Cytarabine; IO, inotuzumab ozogamicin; VIP, Vincristine + Idarubicin + Prednisone; DECM, Decitabine + Cyclophosphamide + Etoposide + Mitoxantrone; CVAD, Cyclophosphamide + Vincristine + Doxorubicin + Prednisone; VCP, Vincristine + Cyclophosphamide + Prednisone; N, not tested.

### 3.3 Adverse reactions

Hematologic adverse events were observed in half of the enrolled patients. Adverse events greater than grade 3 were leukopenia (36.4%), anemia (36.4%), and thrombocytopenia (31.8%). Non-hematologic adverse events were also common, but all were grade <3: elevated aminotransferases (40.9%), hypocalcemia (40.9%), and concurrent fever/pneumonia (31.8%) (Figure. 1H). Two serious adverse events were recorded: one patient experienced cutaneous-mucosal hemorrhage at a venipuncture site, and another patient died from acute myocardial infarction complicated by cerebral infarction.

## 4 Discussion

The prognosis of Ph + ALL has progressively improved from the era of single-agent chemotherapy to the hematopoietic stem cell transplantation (HSCT) era, with significant advancements observed following the introduction of TKIs. However, patients treated with first- or second-generation TKIs often develop drug-resistant mutations, leading to reduced complete molecular response (CMR) rates, increased relapse rates, and poor outcomes. The emergence of ponatinib, a third-generation TKI, has partially addressed the T315I resistance mutation. In a study evaluating ponatinib combined with high-dose CVAD as first-line therapy for Ph + ALL patients over 80 months of follow-up, the estimated 6-year event-free survival rate was 65%, with an overall survival rate of 75% (5). Additionally, a phase 2 trial involving patients aged ≥60 years or those ineligible for intensive chemotherapy and allogeneic HSCT demonstrated that ponatinib combined with prednisone achieved a 40.9% CMR rate at 24 weeks, with a median CMR duration of 11.6 months and median event-free survival (EFS) of 14.3 months (11). Ponatinib has shown significant antileukemic activity in clinical practice. However, its use is associated with a high risk of cardiovascular events, including severe or fatal arterial occlusions and venous thromboembolism, which led to the temporary suspension of sales by the U.S. Food and Drug Administration (FDA) (12). Notably, ponatinib remains unavailable in China. The introduction of Olverembatinib provided a viable treatment option for this disease.

Elias J et al. (13) conducted the first international clinical trial of Olverembatinib in TKI-resistant/intolerant CML and Ph + ALL patients. Among 13 evaluable advanced Ph + ALL cases, 3 (23%) achieved MMR. Two Ph + B-cell precursor ALL patients receiving 30 mg Olverembatinib combined with blinatumomab for one cycle both attained CCyR, with one achieving minimal residual disease (MRD) negativity. This prompted the exploration of Olverembatinib in Ph + ALL. A case series reported four newly diagnosed Ph + ALL adults achieving complete remission (CR) and CCyR after Olverembatinib induction, with 100% 3–6 month disease-free survival and no severe adverse events (14). Another study described five relapsed Ph + ALL patients (15): four achieved CR after 1–2 cycles of Olverembatinib plus chemotherapy, with three attaining CCyR. Li XL et al. (16) treated six pediatric relapsed Ph + ALL cases with Olverembatinib, achieving CR with MRD negativity in four patients after a median 70-day treatment, with no grade 3–4 toxicities. Fan XS et al. (17) combined Olverembatinib with blinatumomab in two relapsed T315I-mutated Ph + ALL patients, both achieving CR and MRD negativity after one cycle.

Zhang XY et al. (18) treated adult patients with Ph + ALL who had refractory/relapsed disease or persistent minimal residual disease (MRD) with Olverembatinib combined with inotuzumab ozogamicin, bridging to hematopoietic stem cell transplantation. With a median follow-up of 564 days, the 2-year overall survival rate and recurrence-free survival rate were 83.3% ± 15.2 and 62.9% ± 17.9%, respectively. Nine patients (64.3%) successfully bridged to HSCT. No cases of veno-occlusive disease (VOD)/sinusoidal obstruction syndrome (SOS) occurred, and the 100-day post-transplantation mortality was 0%. Six of these patients achieved complete molecular response (CMR) before allogeneic HSCT (allo-HSCT).

A prospective clinical study used Olverembatinib combined with venetoclax and reduced-intensity chemotherapy to treat patients with newly diagnosed Ph + ALL (19). Among 79 patients evaluable after 3 cycles of treatment, the regimen achieved a CMR rate of 62.0% at 3 months without the use of intensive chemotherapy or immunotherapy. No induction deaths occurred. With a median follow-up of 12 months, the estimated 1-year OS and EFS rates were 93.1% (95% CI, 86.4–99.8) and 89.1% (95% CI, 80.3–97.9), respectively.

Currently, more clinical studies on Olverembatinib are underway (20), including the phase 3 registrational POLARIS-1 (NCT06051409; in patients with newly diagnosed Philadelphia chromosome-positive acute lymphoblastic leukemia), POLARIS-2 (NCT06423911; in patients with CML with or without the T315I mutation). The results of these clinical trials are expected to provide further evidence for the treatment of Ph + ALL and CML with Olverembatinib. Here, our real-world research reports 22 cases of Ph + ALL patients treated with Olverembatinib: including 12 patients who relapsed after first/s-generation TKI therapies, and 10 patients with persistent pre-transplant MRD positivity and loss of MMR. Of the 22 patients, 16 (72.7%) subsequently achieved minimal residual disease (MRD) negativity, and 7 (31.8%) proceeded to hematopoietic stem cell transplantation (HSCT). In this research, newly diagnosed patients were excluded from the study cohort. Additionally, the relatively small sample size of the included patients represents a limitation of this study. Currently, several relevant clinical trials are underway. Therefore, further research is essential to investigate the efficacy and adverse reactions of Olverembatinib comprehensively.

## 5 Conclusion

Collectively, in relapsed Ph + ALL, Olverembatinib exhibits better efficacy and tolerability. Although adverse event rates remain notable, real-world data indicate that most patients maintain adequate treatment tolerance. Prospective studies are warranted to further validate its clinical utility.

## Data Availability

The raw data supporting the conclusions of this article will be made available by the authors, without undue reservation.
